# Prognostic importance of emerging cardiac, inflammatory, and renal biomarkers in chronic heart failure patients with reduced ejection fraction and anaemia: RED‐HF study

**DOI:** 10.1002/ejhf.988

**Published:** 2017-09-27

**Authors:** Paul Welsh, Lei Kou, Changhong Yu, Inder Anand, Dirk J. van Veldhuisen, Aldo P. Maggioni, Akshay S. Desai, Scott D. Solomon, Marc A. Pfeffer, Sunfa Cheng, Lars Gullestad, Pål Aukrust, Thor Ueland, Karl Swedberg, James B. Young, Michael W. Kattan, Naveed Sattar, John J.V. McMurray

**Affiliations:** ^1^ Institute of Cardiovascular and Medical Sciences University of Glasgow Glasgow UK; ^2^ Department of Quantitative Health Sciences Cleveland Clinic Cleveland OH USA; ^3^ VA Medical Center University of Minnesota Minneapolis MN USA; ^4^ University Medical Center Groningen The Netherlands; ^5^ ANMCO Research Center Florence Italy; ^6^ Cardiovascular Division Brigham and Women's Hospital Boston MA USA; ^7^ Amgen Thousand Oaks CA USA; ^8^ Department of Cardiology Oslo University Hospital Rikshospitalet, Oslo Norway; ^9^ Institute of Clinical Medicine, Faculty of Medicine University of Oslo Oslo Norway; ^10^ Research Institute of Internal Medicine Oslo University Hospital Rikshospitalet Oslo Norway; ^11^ K.G. Jebsen Inflammatory Research Center University of Oslo Oslo Norway; ^12^ Section of Clinical Immunology and Infectious Diseases Oslo University Hospital Rikshospitalet Oslo Norway; ^13^ University of Gothenburg, Gothenburg, Sweden, and National Heart and Lung Institute Imperial College London UK

**Keywords:** Heart failure, Natriuretic peptides, Troponin, Adrenomedullin, Copeptin, Cystatin C

## Abstract

**Aims:**

To test the prognostic value of emerging biomarkers in the Reduction of Events by Darbepoetin Alfa in Heart Failure (RED‐HF) trial.

**Methods and results:**

Circulating cardiac [N‐terminal pro‐B‐type natriuretic peptide (NT‐proBNP), and high‐sensitivity troponin T (hsTnT)], neurohumoral [mid‐regional pro‐adrenomedullin (MR‐proADM) and copeptin], renal (cystatin C), and inflammatory [high‐sensitivity C‐reactive protein (hsCRP)] biomarkers were measured at randomization in 1853 participants with complete data. The relationship between these biomarkers and the primary composite endpoint of heart failure hospitalization or cardiovascular death over 28 months of follow‐up (n = 834) was evaluated using Cox proportional hazards regression, the c‐statistic and the net reclassification index (NRI). After adjustment, the hazard ratio (HR) for the composite outcome in the top tertile of the distribution compared to the lowest tertile for each biomarker was: NT‐proBNP 3.96 (95% CI 3.16–4.98), hsTnT 3.09 (95% CI 2.47–3.88), MR‐proADM 2.28 (95% CI 1.83–2.84), copeptin 1.66 (95% CI 1.35–2.04), cystatin C 1.92 (95% CI 1.55–2.37), and hsCRP 1.51 (95% CI 1.27–1.80). A basic clinical prediction model was improved on addition of each biomarker individually, most strongly by NT‐proBNP (NRI +62.3%, P < 0.001), but thereafter was only improved marginally by addition of hsTnT (NRI +33.1%, P = 0.004). Further addition of biomarkers did not improve discrimination further. Findings were similar for all‐cause mortality.

**Conclusion:**

Once NT‐proBNP is included, only hsTnT moderately further improved risk stratification in this group of chronic heart failure with reduced ejection fraction patients with moderate anaemia. NT‐proBNP and hsTnT far outperform other emerging biomarkers in prediction of adverse outcome.

## Introduction

B‐type natriuretic peptide, produced by the myocardium primarily in response to volume overload and increase in wall stress, and its inactive metabolite N‐terminal pro‐B‐type natriuretic peptide (NT‐proBNP), are established prognostic markers in patients with heart failure and reduced ejection fraction (HFrEF).^1^ Similarly, elevated levels of cardiac troponins reflecting cardiomyocyte necrosis, consistently relate to worse clinical outcomes in both acute^2^, ^3^ and chronic^4^, ^5^, ^6^, ^7^, ^8^ HFrEF patients. Both these biomarkers may provide additive prognostic information to routinely collected demographic, clinical and laboratory data in patients with chronic heart failure (HF).^5^, ^6^, ^7^

The relative or incremental predictive role of other emerging biomarkers related to cardiac function, renal function, neurohormonal activation and inflammation, either individually or as part of a multimarker approach, in HFrEF is less certain. Several potential candidates for inclusion in a multimarker approach, thought to be of pathophysiological importance in HF have been identified. The 52‐amino‐acid peptide adrenomedullin is a long‐acting vasodilator produced by many tissues including cardiovascular system and is increased in HF. Adrenomedullin is unstable in blood and difficult to monitor, whereas its mid‐regional pro‐peptide [mid‐regional pro‐adrenomedullin (MR‐proADM)] is stable, easy to measure and has been found to predict outcome in acute and chronic HF patients.^9^, ^10^, ^11^ C‐terminal pro‐arginine vasopressin (AVP), more commonly known as copeptin, is a stable precursor of AVP, a circulating peptide vasoconstrictor, which is also involved in water homeostasis.^12^ Plasma copeptin concentrations have also been shown, albeit variably, to add incremental prognostic information to both NT‐proBNP and high‐sensitivity troponin T (hsTnT) in the acute and chronic setting.^13^, ^14^, ^15^, ^16^, ^17^, ^18^ In addition to cardiac biomarkers, renal function is also an established and powerful predictor of outcomes in HFrEF.^1^ Although most commonly evaluated by estimation of glomerular filtration rate (eGFR) using several creatinine‐based formulae, the low‐molecular‐weight peptide cystatin C may be a more precise measure of renal function in particular patient groups. In acute and chronic HF, cystatin C has been reported to be a better predictor of adverse outcomes than creatinine‐based eGFR, and to add incremental prognostic information to both NT‐proBNP and hsTnT.^19^, ^20^, ^21^, ^22^, ^23^ Finally, whereas inflammation has been implicated in the pathogenesisis of HF, the relative value of high‐sensitivity C‐reactive protein (hsCRP), a reliable and stable marker of systemic inflammation, as a prognostic marker in patients with HFrEF is uncertain.^7^, ^24^, ^25^, ^26^ The main questions about emerging biomarkers in HF prognostication are: (i) how do they perform as prognostic markers compared to B‐type natriuretic peptides, troponin or both (i.e. could we find a better replacement for one or both of these effective prognostic markers?), or (ii) do they individually or collectively add further meaningful prognostic information to the routinely collected variables including a B‐type natriuretic peptide and a troponin? Few studies have addressed these questions for the emerging biomarkers used either alone or in a multiple biomarker panel.

Therefore, the primary aim of this study was to assess the prognostic value of several promising emerging cardiac, neurohormonal, renal and inflammatory biomarkers beyond that provided by NT‐proBNP, troponin, and hsTnT individually and in combination, in patients enrolled in the Reduction of Events by Darbepoetin Alfa in Heart Failure (RED‐HF) trial.

## Methods

### Study design and patient selection

RED‐HF was a randomized placebo‐controlled double‐blind trial designed to test the effect of treatment with darbepoetin alfa, targeting a haemoglobin of 13.0 g/dL, on clinical outcomes in patients with HFrEF and anaemia.^27^, ^28^ Participating patients had to be ≥18 years, New York Heart Association (NYHA) class II–IV (NYHA class II patients had to have an unplanned hospital admission or emergency room visit for a cardiovascular reason within 12 months prior to randomization), left ventricular ejection fraction (LVEF) ≤40% with HFrEF diagnosed for ≥3 months, had haemoglobin in the range of 90–120 g/L, and receiving stable optimal HF therapy. Exclusion criteria included transferrin saturation < 15%, evidence of bleeding or other correctable causes of anaemia, creatinine >265 μmol/L (>3.0 mg/dL), and uncontrolled hypertension (>160/100 mmHg). The study randomized 2278 patients to either darbepoetin alfa (*n* = 1136) or placebo (*n* = 1142) at 453 sites in 33 countries between June 2006 and May 2012 with a median follow‐up of 28 months. The three regions with largest enrolment were North America (*n* = 644), Western Europe (*n* = 609), and Central/Eastern Europe (*n* = 454). The ethics committee at each study centre approved the trial design, and patients provided written informed consent, including storage of blood samples for future biomarker analysis.

### Outcomes

The primary outcome for the RED‐HF trial was the composite of death from any cause or first hospitalization for worsening HF. The pre‐specified outcomes investigated in the present post‐hoc analysis were (i) the composite cardiovascular death outcome of first hospitalization for worsening HF or death from cardiovascular causes, and (ii) all‐cause mortality. The focus on cause‐specific mortality outcome has been discussed in a recent review.^29^ Outcomes were adjudicated according to pre‐specified definitions by an independent committee blinded to treatment assignment.^28^

### Study assessments and biomarker assays

At randomization, fasting venous blood was collected and serum and plasma were separated and stored at –80 °C until thawing for assay. Serum and plasma samples were shipped on dry ice to a central laboratory (University of Glasgow) for assay of six biomarkers in a single batch. We did not specifically test frozen stability of the biomarkers, but all assays were conducted on first thaw. MR‐proADM and copeptin were measured in plasma and NT‐proBNP, hsTnT, cystatin C, and hsCRP were measured in serum using automated clinical platforms using manufacturer calibrators and controls. MR‐proADM and ultra‐sensitive copeptin assays were performed (using a single batch number) on a Kryptor plus (ThermoFisher Scientific, Hemel Hempstead, UK), assays had limits of detection of 0.05 nmol/L and 0.9 pmol/L, and had low and high control coefficients of variation of 6.4%, 5.3% and 6.9%, 11.8%, respectively. NT‐proBNP and hsTnT were measured using an e411 (Roche Diagnostics, Burgess Hill, UK), assays had limits of detection of 5 ng/L (5 pg/mL) and 3 ng/L, respectively, and had low and high control coefficients of variation (day to day) of 6.4%, 6.2% and 6.5%, 3.7%, respectively. Cystatin C and CRP were measured using a c311 (Roche Diagnostics), assays had limits of detection of 0.4 mg/L and 0.1 mg/L, respectively, and had control coefficients of variation of 2.8% and 3.9%, respectively. Controls for all biomarkers were of a single lot number for the entire study.

### Statistical analysis

Baseline characteristics of participants were tabulated by composite outcome group using means and standard deviations for continuous variables, or using median and interquartile range for variables with skewed distribution, and numbers and percentages for categorical variables. Comparisons between biomarker concentrations in outcome groups were made using two tailed *t*‐tests for normally distributed biomarkers, and Wilcoxon rank sum test for non‐normally distributed biomarkers. The relationships among baseline circulating biomarkers were tested using Spearman correlations, and for other clinical risk factors trends across tertiles of the biomarker distribution were assessed using chi‐square tests, one‐way analysis of variance, or Kruskal–Wallis rank sum test depending on variable type and distribution.

Kaplan–Meier survival curves were constructed to illustrate time‐to‐event outcomes of patients according to tertiles of biomarker distribution at baseline and compared using the log‐rank test. The associations between baseline levels of biomarkers and outcomes were evaluated using multivariable Cox proportional hazard models, adjusting for region, age, sex, race, body mass index, smoking, systolic and diastolic blood pressure, diabetes, chronic obstructive pulmonary disease, NYHA class, LVEF, time since diagnosis, angiotensin‐converting enzyme (ACE) inhibitor/angiotensin receptor blocker (ARB) use, beta‐blocker use, creatinine, HF hospitalization within last 6 months, HF aetiology, stroke, atrial fibrillation/flutter, and heart rate. Diuretics were used in >90% of participants, and were therefore not included in the adjustment models. A total of 1853 patients had complete biomarkers and clinical variable data for analyses. Biomarker utility in predicting outcome was tested using the fully adjusted model above as a basic comparator clinical prediction model, to which biomarkers were then added. Model discrimination was tested using Harrell's c‐statistic^30^ and the continuous net reclassification index (NRI),^31^ adapted for use in survival models.^32^ For model comparison, c‐statistic and NRI were generated using ordinary non‐parametric bootstrapping, and then *P*‐values obtained from paired *t*‐tests. All statistical analyses were performed using R version 3.2.3 with additional packages of rms, pec and survIDINRI.

## Results

### Baseline characteristics

NT‐proBNP, hsTnT, copeptin, MR‐proADM, cystatin C and hsCRP measurements were made at baseline in 1941, 1946, 1873, 1878, 1946, and 1946 patients, respectively. All participants had detectable NT‐proBNP and <1% patients had hsTnT <3 ng/L (the assay limit of blank). Biomarker and clinical measurements were complete in 1853 participants. Supplementary material online, *Tables* S1–S6 show the baseline characteristics of the patients by tertiles of the biomarker level. Higher biomarker levels were generally associated measures of HF severity including higher NYHA class, more co‐morbidities, older age and greater impairment of renal function. Supplementary material online, *Table* S7 shows associations between the biomarkers and several clinical variables and amongst themselves. Particularly strong associations were found between all biomarkers and creatinine. NT‐proBNP was also strongly associated with proADM, copeptin, cystatin C.

### Follow‐up for incident events

Over the median 28 months, 1019 patients experienced one of the components of the composite outcome of first HF hospitalization or cardiovascular death and 932 participants died from any cause (834 composite events and 769 all‐cause deaths in those with complete biomarker data). Those who experienced the composite outcome during follow‐up were older, more likely to be male, white, and smokers. They were also more likely to have a history of stroke, chronic obstructive pulmonary disease, atrial fibrillation and features of worse HF (higher NYHA class, lower LVEF, lower systolic blood pressure, longer duration of disease), and also had higher serum creatinine (*Table* 1). They were less likely to be treated with an ACE inhibitor or ARB (*Table* 1). Baseline concentrations of all the biomarkers of interest were elevated in those who experienced the composite HF event, and in particular there was an approximate three‐fold difference in circulating levels of NT‐proBNP (median 3067 vs. 1027, *P* < 0.001) (*Table* 1). Crude associations were similar for the all‐cause mortality endpoint (supplementary material online, *Table* S8).

**Table 1:** Baseline characteristics of RED‐HF participants by whether or not a composite cardiovascular death/heart failure hospitalization occurred during follow‐up

Characteristic	HF hospitalization or CV death (*n* = 1019)	No HF hospitalization or CV death (*n* = 1259)	*P*‐value
Age, years	71.4 (10.9)	68.4 (11.7)	<0.001
Male sex	675 (66.2)	659 (52.3)	<0.001
Race			<0.001
White	765 (75.1)	784 (62.3)	
Black	94 (9.2)	108 (8.6)	
Other	160 (15.7)	367 (29.1)	
BMI, kg/m^2^	25.9 (23.3–29.7)	26.5 (23.5–30.4)	0.055
Smoking			<0.001
Current	52 (5.1)	45 (3.6)	
Former	446 (43.8)	401 (31.9)	
Never	520 (51.1)	811 (64.5)	
Systolic BP, mmHg	117 (19)	122 (17)	<0.001
Diastolic BP, mmHg	67.3 (10.9)	71.1 (10.6)	<0.001
Diabetes	496 (48.7)	559 (44.4)	0.042
Previous stroke	98 (9.6)	81 (6.4)	0.005
COPD	214 (21.0)	153 (12.2)	<0.001
Atrial fibrillation/flutter	431 (42.3)	316 (25.1)	<0.001
NYHA class			<0.001
II	280 (27.5)	511 (40.6)	
III/IV	739 (72.5)	748 (59.4)	
LVEF, %	29.2 (7.1)	31.2 (6.5)	<0.001
Ischaemic aetiology	793 (77.8)	868 (68.9)	<0.001
HF duration, years	4.4 (1.7–8.5)	3.0 (1.1–7.1)	<0.001
Heart rate, b.p.m.	72.1 (11.7)	71.8 (10.7)	0.546
Beta‐blocker use	854 (83.8)	1083 (86.0)	0.141
ACE‐I or ARB use	880 (86.4)	1145 (90.9)	<0.001
Creatinine, mg/dL	1.5 (1.2–2.0)	1.2 (1.0–1.6)	<0.001
NT‐proBNP, ng/L	3067 (1458–6615)	1027 (324–2541)	<0.001
Troponin T, ng/L	35.6 (22.2–53.0)	19.1 (11.2–31.9)	<0.001
MR‐proADM, nmol/L	1.1 (0.8–1.5)	0.8 (0.6–1.1)	<0.001
Copeptin, pmol/L	20.3 (9.4–36.0)	11.0 (5.4–22.8)	<0.001
Cystatin C, mg/L	1.8 (0.7)	1.4 (0.7)	<0.001
CRP, mg/L	3.5 (1.3–8.3)	2.2 (0.9–5.6)	<0.001

Values are mean (standard deviation), median (interquartile range), or number (%).

ACE‐I, angiotensin‐converting enzyme inhibitor; ARB, angiotensin receptor blocker; BMI, body mass index; BP, blood pressure; COPD, chronic obstructive pulmonary disease; CRP, C‐reactive protein; CV, cardiovascular; HF, heart failure; LVEF, left ventricular ejection fraction; NT‐proBNP, N‐terminal pro‐B‐type natriuretic peptide; MR‐proADM, mid‐regional pro‐adrenomedullin.

### Association between biomarker concentrations and incident events

**Figure 1:**
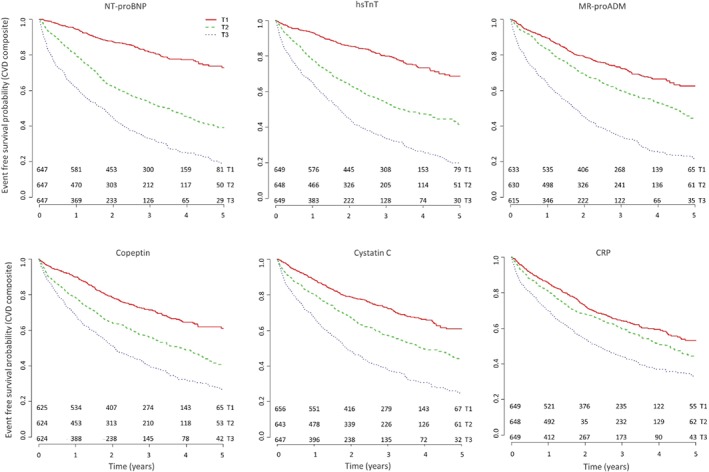
Event‐free survival experiences of the participants by tertiles of the biomarker distribution corresponding to cut‐offs of 947, 3067 ng/L for N‐terminal pro‐B‐type natriuretic peptide (NT‐proBNP), 17.9, 36.2 ng/L for high‐sensitivity troponin T (hsTnT), 0.73, 1.14 nmol/L for mid‐regional pro‐adrenomedullin (MR‐proADM), 8.66, 22.99 pmol/L for copeptin, 1.22, 1.84 mg/L for cystatin C, and 1.40, 4.94 mg/L for C‐reactive protein (CRP). All trends are log‐rank P < 0.001. CVD, cardiovascular death.

The unadjusted Kaplan–Meier curves (*Figure* 1) showed that the risk of the composite outcome was higher in patients with higher levels of each biomarker (log‐rank *P* < 0.001 for all).

**Figure 2:**
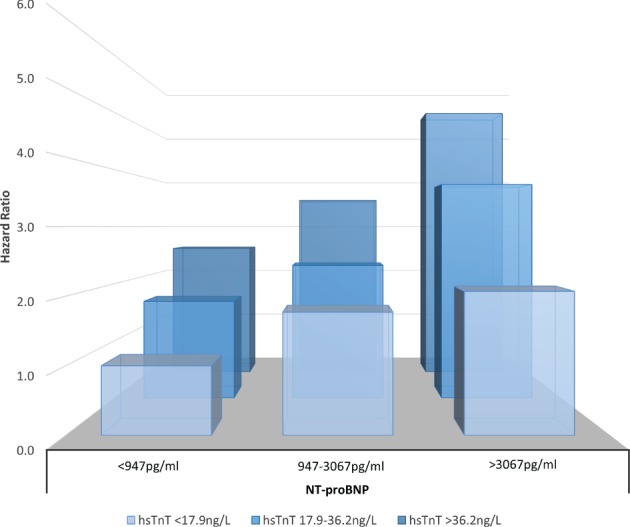
Association of N‐terminal pro‐B‐type natriuretic peptide (NT‐proBNP) and high‐sensitivity troponin T (hsTnT) with primary outcome by tertiles of the distribution of each biomarker, after adjustment for classical risk factors (model as in Table 2).

In the Cox regression analysis, after adjusting for other risk factors in the clinical model, NT‐proBNP was still strongly associated with risk of the composite endpoint both as a continuous variable and by tertiles of the distribution. Patients in the highest tertile of the NT‐proBNP distribution had an approximately four‐fold higher risk of the composite outcome compared to those in the lowest tertile (*Table* 2). For other biomarkers, the higher risk in the top tertile of the distribution ranged between 1.5‐fold for hsCRP and three‐fold for hsTnT (*Table* 2). A combination of NT‐proBNP and hsTnT gave the strongest risk prediction compared to combinations of other markers and after adjustment, i.e., those in the top tertile for both NT‐proBNP and hsTnT were at 5.3‐fold higher risk compared to those in the lowest tertile of the distribution for both biomarkers (*Figure* 2).

**Table 2:** Adjusted hazard ratio (95% confidence interval) of the primary endpoint and all‐cause mortality in relation to baseline biomarkers, by tertiles and by 1 standard deviation increase in log‐transformed biomarkers (n = 1856)

	*N* participants (*n* HF/CV death, *n* all‐cause death)	HF hospitalization or CV death	All‐cause mortality
NT‐proBNP			
T1	610 (119, 124)	Ref.	Ref.
T2	624 (299, 257)	2.54 (2.04–3.17)	1.84 (1.47–2.31)
T3	619 (416, 388)	3.96 (3.16–4.98)	2.98 (2.38–3.74)
per 1 SD	1853 (834, 769)	1.91(1.74–2.10) *P* < 0.001	1.80 (1.63–1.99) *P* < 0.001
Troponin T			
T1	618 (141, 127)	Ref.	Ref.
T2	621 (288, 256)	2.06 (1.66–2.55)	1.90 (1.51–2.38)
T3	614 (405, 386)	3.09 (2.47–3.88)	3.10 (2.45–3.93)
per 1 SD	1853 (834, 769)	1.60 (1.48–1.73) *P* < 0.001	1.61 (1.48–1.76) *P* < 0.001
MR‐proADM			
T1	628 (182, 157)	Ref.	Ref.
T2	620 (269, 251)	1.30 (1.07–1.59)	1.40 (1.13–1.73)
T3	605 (383, 361)	2.28 (1.83–2.84)	2.49 (1.96–3.15)
per 1 SD	1853 (834, 769)	1.53 (1.39–1.68) *P* < 0.001	1.63(1.47–1.80) *P* < 0.001
Copeptin			
T1	619 (187, 175)	Ref.	Ref.
T2	619 (282, 249)	1.46 (1.2–1.76)	1.42 (1.16–1.73)
T3	615 (365, 345)	1.66 (1.35–2.04)	1.78 (1.44–2.20)
per 1 SD	1853 (834, 769)	1.25 (1.15–1.36) *P* < 0.001	1.31 (1.19–1.43) *P* < 0.001
Cystatin C			
T1	633 (188, 169)	Ref.	Ref.
T2	613 (278, 253)	1.36 (1.12–1.66)	1.28 (1.04–1.57)
T3	607 (368, 347)	1.92 (1.55–2.37)	1.90 (1.52–2.37)
per 1 SD	1853 (834, 769)	1.36 (1.23–1.50) *P* < 0.001	1.41 (1.27–1.56) *P* < 0.001
CRP			
T1	621 (228, 210)	Ref.	Ref.
T2	614 (268, 245)	1.21 (1.01–1.44)	1.23 (1.02–1.48)
T3	618 (338, 314)	1.51 (1.27–1.80)	1.58 (1.32–1.90)
per 1 SD	1853 (834, 769)	1.17 (1.09–1.25) *P* < 0.001	1.19 (1.1–1.28) *P* < 0.001

CRP, C‐reactive protein; CV, cardiovascular; HF, heart failure; MR‐proADM, mid‐regional pro‐adrenomedullin; NT‐proBNP, N‐terminal pro‐B‐type natriuretic peptide; SD, standard deviation.

Model: adjusting for region, age, sex, race, body mass index, smoking, systolic and diastolic blood pressure, diabetes, chronic obstructive pulmonary disease, New York Heart Association class, left ventricular ejection fraction, time since diagnosis, angiotensin‐converting enzyme inhibitor/angiotensin receptor blocker use, beta‐blocker use, creatinine, HF hospitalization within last 6 months, HF aetiology, stroke, atrial fibrillation/flutter, heart rate.

The association between higher concentrations of all biomarkers and risk of all‐cause mortality was similar to their prediction of the composite cardiovascular death endpoint. Again, NT‐proBNP and hsTnT were the strongest predictors (*Table* 2).

Study treatment did not modify the relationship between baseline biomarker concentrations and outcomes (data not shown).

### Incremental predictive information from biomarkers individually and in combination

The basic clinical risk‐prediction model for the composite endpoint yielded a c‐index of 0.687. Each biomarker improved discrimination when added individually to the clinical model. When added to the basic model one at a time, NT‐proBNP caused the largest increase in c‐index for a single biomarker (+0.045, *P* < 0.001), followed by hsTnT (+0.028, *P* < 0.001), MR‐proADM (+0.017, *P* < 0.001), cystatin C (+0.01, *P* < 0.001), copeptin (+0.008, *P* < 0.001), and hsCRP (+0.005, *P* = 0.056) (*Table* 3). When NT‐proBNP was included in the basic risk model, none of the other biomarkers improved discrimination further. However, hsTnT added to NT‐proBNP improved NRI. Adding NT‐proBNP to the basic clinical model (+62.3%, *P* < 0.001) improved classification of both cases and controls, and addition of hsTnT further enhanced the NRI (+33.1%, *P* = 0.004). Adding other biomarkers made no further improvements.

**Table 3:** C‐index and continuous net reclassification index for heart failure hospitalization or cardiovascular death using classical risk markers plus biomarkers (continuous) in those with complete data (n = 1853, n events 834)

Biomarker	C‐index (95% CI) *P*‐value		Net reclassification index		
	Classical markers	Classical + NT‐proBNP	Group	Classical markers	Classical + NT‐proBNP
Comparator	0.687	0.732	–	–	–
	(0.668–0.706)	(0.714–0.751)			
NT‐proBNP	0.732	–	Cases	28.9%	–
	(0.714–0.751)		Non‐case	33.3%	–
	*P* < 0.001		Overall	62.3%, *P* < 0.001	–
Troponin T	0.715	0.739	Cases	17.1%	9.8%
	(0.697–0.734)	(0.723–0.756)	Non‐case	35.8%	23.3%
	*P* < 0.001	*P* = 0.274	Overall	52.9%, *P* < 0.001	33.1%, *P* = 0.004
MR‐proADM	0.704	0.735	Cases	21.8%	11.5%
	(0.685–0.723)	(0.718–0.751)	Non‐case	24.5%	8.2%
	*P* < 0.001	*P* = 0.645	Overall	46.3%, *P* < 0.001	19.7%, *P* = 0.132
Copeptin	0.695	0.735	Cases	13.5%	10.9%
	(0.677–0.714)	(0.718–0.752)	Non‐case	6.9%	–3.1%
	*P* < 0.001	*P* = 0.617	Overall	20.4%, *P* = 0.034	7.8%, *P* = 0.36
Cystatin C	0.697	0.734	Cases	17.1%	9.9%
	(0.679–0.711)	(0.718–0.752)	Non‐case	8.2%	–8.2%
	*P* < 0.001	*P* = 0.679	Overall	25.3%, *P* = 0.006	1.7%, *P* = 0.821
CRP	0.692	0.734	Cases	6.5%	4.8%
	(0.673–0.711)	(0.718–0.751)	Non‐case	5.7%	–4.4%
	*P* = 0.056	*P* = 0.663	Overall	12.2%, *P* = 0.158	0.4%, *P* = 0.99
All biomarkers	0.738	0.738	Cases	29.1%	11.9%
	(0.722–0.755)	(0.722–0.755)	Non‐case	40.9%	19.5%
	*P* < 0.001	*P* = 0.332	Overall	70%, *P* < 0.001	31.4%, *P* = 0.008

CI, confidence interval; CRP, C‐reactive protein; MR‐proADM, mid‐regional pro‐adrenomedullin; NT‐proBNP, N‐terminal pro‐B‐type natriuretic peptide.

Model: adjusting for region, age, sex, race, body mass index, smoking, systolic and diastolic blood pressure, diabetes, chronic obstructive pulmonary disease, New York Heart Association class, left ventricular ejection fraction, time since diagnosis, angiotensin‐converting enzyme inhibitor/angiotensin receptor blocker use, beta‐blocker use, creatinine, heart failure hospitalization within last 6 months, heart failure aetiology, stroke, atrial fibrillation/flutter, heart rate.

Patterns for prediction of all‐cause mortality were broadly similar. All biomarkers (except hsCRP) improved discrimination individually. NT‐proBNP improved discrimination and NRI most strongly, and only addition of hsTnT resulted in an improvement in NRI (*Table* 4).

**Table 4:** C‐index and continuous net reclassification index for all‐cause death using classical risk markers plus biomarkers (continuous) in those with complete data (n = 1853, n events 769)

Biomarker	C‐index (95% CI) *P*‐value		Net reclassification index		
	Classical markers	Classical + NT‐proBNP	Group	Classical markers	Classical + NT‐proBNP
Comparator	0.669	0.713	–	–	–
	(0.651–0.688)	(0.694–0.732)			
NT‐proBNP	0.713	–	Cases	23.7%	–
	(0.694–0.732)		Non‐case	22.6%	–
	*P* = 0.002		Overall	46.3%, *P* < 0.001	–
Troponin T	0.699	0.721	Cases	14.8%	6.3%
	(0.680–0.718)	(0.704–0.738)	Non‐case	31.7%	20.6%
	*P* = 0.002	*P* = 0.196	Overall	46.5%, *P* < 0.001	26.9%, *P* = 0.01
MR‐proADM	0.687	0.714	Cases	18.0%	9.5%
	(0.668–0.706)	(0.695–0.731)	Non‐case	24.6%	6.5%
	*P* = 0.002	*P* = 0.890	Overall	42.6%, *P* < 0.001	16%, *P* = 0.13
Copeptin	0.676	0.715	Cases	12.8%	11.2%
	(0.658–0.695)	(0.697–0.732)	Non‐case	6.5%	–2.5%
	*P* = 0.014	*P* = 0.740	Overall	19.3%, *P* = 0.036	8.7%, *P* = 0.346
Cystatin C	0.679	0.715	Cases	18.7%	10.7%
	(0.661–0.698)	(0.698–0.732)	Non‐case	4.5%	–10.6%
	*P* = 0.002	*P* = 0.726	Overall	23.2%, *P* = 0.006	0.2%, *P* = 0.635
CRP	0.673	0.714	Cases	7.3%	5.7%
	(0.654–0.692)	(0.696–0.732)	Non‐case	6.5%	–5.5%
	*P* = 0.160	*P* = 0.778	Overall	13.8%, *P* = 0.1	0.1%, *P* = 0.783
All biomarkers	0.719	0.719	Cases	24.1%	11.7%
	(0.701–0.737)	(0.701–0.737)	Non‐case	36.7%	15.6%
	*P* = 0.002	*P* = 0.306	Overall	60.7%, *P* < 0.001	27.2%, *P* < 0.001

CI, confidence interval; CRP, C‐reactive protein; MR‐proADM, mid‐regional pro‐adrenomedullin; NT‐proBNP, N‐terminal pro‐B‐type natriuretic peptide.

Model: adjusting for region, age, sex, race, body mass index, smoking, systolic and diastolic blood pressure, diabetes, chronic obstructive pulmonary disease, New York Heart Association class, left ventricular ejection fraction, time since diagnosis, angiotensin‐converting enzyme inhibitor/angiotensin receptor blocker use, beta‐blocker use, creatinine, heart failure hospitalization within last 6 months, heart failure aetiology, stroke, atrial fibrillation/flutter, heart rate.

## Discussion

In this analysis, we evaluated four emerging biomarkers in addition to NT‐proBNP and hsTnT in one of the largest studies of chronic HF patients to date. Although all biomarkers improved risk stratification when added to the basic clinical model, NT‐proBNP outperformed all the other biomarkers in improving model discrimination. Moreover, apart from hsTnT, none of the other biomarkers improved model discrimination when added to NT‐proBNP.

Several established risk models in HF, based on routinely collected clinical data, perform reasonably effectively.^33^, ^34^ In recent years, there has been an explosion of reports of new biomarkers in HF, many of which individually predict adverse outcomes in HF.^2^, ^3^, ^4^, ^5^, ^6^, ^7^, ^8^, ^9^, ^10^, ^11^, ^12^, ^13^, ^14^, ^15^, ^16^, ^17^, ^18^, ^19^, ^20^, ^21^, ^22^, ^23^, ^24^, ^25^, ^35^ However, since both NT‐proBNP and high‐sensitivity troponins are available as standardized assays in most countries and are routinely used in the diagnostic work‐up of HF and myocardial infarction, these findings have important implications for usual clinical practice.

Conceptually, a multimarker model is attractive in HF because an appropriate selection of biomarkers should better reflect the complex pathophysiology of this syndrome. While our biomarker panel reflected neurohumoral pathways (copeptin, MR‐proADM), renal function (cystatin C) and inflammation (hsCRP), these pathways may offer redundant clinical information, and other potentially important pathophysiological processes such as matrix remodelling and oxidative stress were not encompassed by our panel. Moreover, although hsCRP is a reliable marker of inflammation, it may not capture information from all relevant upstream inflammatory processes in vascular and myocardial diseases. Therefore, we cannot rule out that more specific inflammatory markers or alternative biomarkers such as ST2, galectin‐3 and urinary isoprostanes, might have been of additional value. However, our data suggest that novel biomarkers even moderately correlated with NT‐proBNP and hsTnT are unlikely to provide meaningful additional risk prediction. Moreover, although our panel of biomarkers could reflect several pathogenic pathways involved in the development of HF, it must be recognized that it is not always clear what pathophysiological mechanism or mechanisms lead to increased levels of a particular biomarker and it may be overly simplistic to categorize individual biomarkers in HFrEF in this way. As such, our data suggest that multimarker approaches to HF risk stratification are only likely to be worthwhile where the biomarkers included provide information about pathways distinct from that provided by natriuretic peptides or troponins. This is no small consideration; troponins and natriuretic peptides might largely ‘capture’ information from not only cardiac, but also, neurohumoral, renal, and inflammatory pathways (as demonstrated by their strong inter‐associations), which is partly what makes them effective biomarkers in risk prediction in a range of populations.

Our study has several limitations. We used a clinical trial cohort, therefore although the patients are more homogeneous than in unselected cohorts, as such the data may not be generalizable to other chronic HF cohorts. There are some regional differences in patient characteristics, but we adjusted for region in our Cox models. The majority of our patients had relatively advanced HFrEF, although it is in this group that prognostication may be most relevant. Our patients also had anaemia, although this was mild (median haemoglobin 112 g/dL) and anaemia is common in HFrEF, especially in more advanced cases. Despite this, our findings are broadly in line with, and expand on, recently published data from an unselected cohort of HF patients.^35^ The narrow range of haemoglobin concentrations among participants precludes meaningful study of the way haemoglobin level might modify the association between other biomarkers and outcomes. The study focuses on a single baseline measure of biomarkers at an arbitrary point in an established chronic disease (i.e. at study recruitment); HFrEF patients may often have clinical blood tests during acute episodes, which is a different setting.

In conclusion, the established biomarker NT‐proBNP offered greatest prognostic utility for adverse outcome in these chronic HFrEF patients with moderate anaemia. Additional neurohumoral, renal, and inflammatory biomarkers did not predict adverse outcome as strongly, and did not add to a basic clinical prediction model which included NT‐proBNP, although incremental information was added by hsTnT. These data strongly suggest that, given their increasing availability and standardized methods for detection in biochemistry departments, future studies in HFrEF must include both NT‐proBNP and high‐sensitivity troponin as benchmarks beyond which other biomarkers, and panels of biomarkers, need to be tested. More work is now also needed to examine potential roles of NT‐proBNP and/or hsTnT in biomarker‐guided therapy in HFrEF.

### Acknowledgements

We thank Elaine Butler, Lynne Cherry, and Sara Jane Duffus, University of Glasgow for technical assistance.

### Funding

Amgen funded the RED‐HF trial. Amgen funded RED‐HF sample storage and biomarker measurement in the cohort (P.W., N.S. and J.J.V.M.). P.W. was funded by British Heart Foundation Fellowship FS/12/62/29889.

**Conflict of interest:** P.W.: grants from Amgen, British Heart Foundation, and Chief Scientist Office. I.A., D.J.v.V., J.B.Y.: members of the RED‐HF Executive Committee (no payments in the last 12 months). M.A.P.: member of the RED‐HF Clinical Endpoint Committee and Executive Committee (no payments in the last 12 months); grants from Novartis and Sanofi; consulting fees from AstraZeneca, Bayer, Boehringer Ingelheim, DalCor, Gilead, GalaxoSmithKline, Janssen, Lilly USA, The Medicines Company, Merck, Novartis, Novo Nordisk, Relypsa, Sanofi, Thrasos, Genzyme and Teva; The Brigham and Women's Hospital has patents for the use of inhibitors of the renin–angiotensin system in selected survivors of myocardial infarction with Novartis. M.A.P. is a co‐inventor; his share of the licensing agreement is irrevocably transferred to charity. S.C.: employee of Amgen. A.P.M.: Trial Committee for Novartis, Cardiorentis, Bayer, Servier. K.S.: consulting fees from AstraZeneca, Amgen, Novartis, Servier, Vifor Pharma; member of the RED‐HF Executive Committee (no payments in the last 12 months). S.D.S.: research grants from Amgen and Novarits; consulting fees from Amgen, Novartis, Bayer, Cytokinetics. A.S.D.: grant to the institution to support endpoint adjudication activities from Amgen; grant from Novartis; consulting fees from Novartis, St Jude/Abbott, Relypsa, Janssen, AstraZeneca, Sanofi, Cheetah Medical. M.W.K.: research grant from Amgen. N.S.: consulting, speaking, and/or honoraria from Amgen, Roche, UCB, Merck, Sanofi/Regeneron, Janssen; grants from Amgen, Chief Scientist Office. J.J.V.M.: consulting fees from Cytokinetics/ Amgen; grant from Amgen. All other authors have no conflict of interest.

## Supporting information


**Table S1.** Baseline characteristics of RED‐HF participants with complete biomarker data by thirds of NT‐proBNP.
**Table S2.** Baseline characteristics of RED‐HF participants with complete biomarker data by thirds of hsTnT.
**Table S3.** Baseline characteristics of RED‐HF participants with complete biomarker data by thirds of MR‐proADM.
**Table S4.** Baseline characteristics of RED‐HF participants with complete biomarker data by thirds of copeptin.
**Table S5.** Baseline characteristics of RED‐HF participants with complete biomarker data by thirds of cystatin C.
**Table S6.** Baseline characteristics of RED‐HF participants with complete biomarker data by thirds of hsCRP.
**Table S7.** Spearman correlation (r) of continuous variables with each other at baseline.
**Table S8.** Baseline characteristics of RED‐HF participants with complete biomarker data by whether or not all‐cause mortality occurred during follow‐up.
