# Supplementary material for: Prognostic importance of emerging cardiac, inflammatory, and renal biomarkers in chronic heart failure patients with reduced ejection fraction and anaemia: RED‐HF study
Source: Eur J Heart Fail. 2017 Sep 27;20(2):268–77. doi: 10.1002/ejhf.988 (PMC6607475; doi:10.1002/ejhf.988)
Supplement: Supplementary file 1 — Table S1. Baseline characteristics of RED‐HF participants with complete biomarker data by thirds of NT‐proBNP. Table S2. Baseline characteristics of RED‐HF participants with complete biomarker data by thirds of hsTnT. Table S3. Baseline characteristics of RED‐HF participants with complete biomarker data by thirds of MR‐proADM. Table S4. Baseline characteristics of RED‐HF participants with complete biomarker data by thirds of copeptin. Table S5. Baseline characteristics of RED‐HF participants with complete biomarker data by thirds of cystatin C. Table S6. Baseline characteristics of RED‐HF participants with complete biomarker data by thirds of hsCRP. Table S7. Spearman correlation (r) of continuous variables with each other at baseline. Table S8. Baseline characteristics of RED‐HF participants with complete biomarker data by whether or not all‐cause mortality occurred during follow‐up. [file EJHF-20-268-s001.docx]

**SUPPLEMENTARY MATERIAL**

**Table S1** **Baseline characteristics of RED-HF participants with complete biomarker data by thirds of NT-proBNP**

| **Characteristic** | **T1 (<947ng/L)** | **T2 (947-3067ng/L)** | **T3 (>3067ng/L)** | **p-value** |
| --- | --- | --- | --- | --- |
|  |  |  |  |  |
| Age (years) | 66.4 (11.6) | 70.4 (11.4) | 71.6 (11) | <0.001 |
| Male sex | 301 (46.5%) | 389 (60.1%) | 444 (68.6%) | <0.001 |
| Race |  |  |  | <0.001 |
| -White | 383 (59.2%) | 438 (67.7%) | 485 (75.0%) |  |
| -Black | 71 (11.0%) | 57 (8.8%) | 41 (6.3%) |  |
| -Other | 193 (29.8%) | 152 (23.5%) | 121 (18.7%) |  |
| BMI (kg/m^2^) | 27.3 (24.7-31.6) | 26.4 (23.2-30.4) | 24.9 (22.4-27.9) | <0.001 |
| Smoking |  |  |  | <0.001 |
| -Current | 26 (4.0%) | 31 (4.8%) | 32 (5.0%) |  |
| -Former | 165 (25.5%) | 257 (39.8%) | 290 (44.9%) |  |
| -Never | 456 (70.5%) | 358 (55.4%) | 324 (50.2%) |  |
| Systolic BP (mmHg) | 123.2 (15.6) | 118.7 (18.3) | 116.7 (18.7) | <0.001 |
| Diastolic BP (mmHg) | 72.2 (10.4) | 67.9 (10.8) | 67.7 (10.6) | <0.001 |
| Diabetes | 281 (43.4%) | 306 (47.3%) | 293 (45.3%) | 0.377 |
| Previous stroke | 39 (6.0%) | 57 (8.8%) | 55 (8.5%) | 0.123 |
| COPD | 91 (14.1%) | 106 (16.4%) | 115 (17.8%) | 0.186 |
| Atrial fibrillation | 126 (19.5%) | 219 (33.8%) | 279 (43.1%) | <0.001 |
| NYHA class |  |  |  | <0.001 |
| -II | 252 (38.9%) | 254 (39.3%) | 170 (26.3%) |  |
| -III/IV | 395 (61.1%) | 393 (60.7%) | 477 (73.7%) |  |
| LVEF % | 32.0 (6.2) | 30.1 (6.7) | 28.5 (7.2) | <0.001 |
| Ischemic etiology | 419 (64.8%) | 502 (77.6%) | 504 (77.9%) | <0.001 |
| HF duration (years) | 3.4 (1.1-7.4) | 3.6 (1.3-7.6) | 3.8 (1.4-7.8) | 0.188 |
| Heart rate (bpm) | 72.6 (10.5) | 71.0 (11.3) | 72.5 (11.8) | 0.012 |
| Beta-blocker use | 534 (82.5%) | 556 (85.9%) | 551 (85.2%) | 0.207 |
| ACE/ARB use | 603 (93.2%) | 583 (90.1%) | 548 (84.7%) | <0.001 |
| Creatinine (mg/dl) | 1.1 (0.9-1.5) | 1.4 (1.1-1.8) | 1.6 (1.3-2.1) | <0.001 |

Values are mean (SD), median (IQR), or percentage. BMI, body mass index; BP, blood pressure; COPD, chronic obstructive pulmonary disease; LVEF, left ventricular ejection fraction.

**Table S2 Baseline characteristics of RED-HF participants with complete biomarker data by thirds of hsTnT**

| **Characteristic** | **T1 (<17.9ng/L)** | **T2 (17.9-36.2ng/L)** | **T3 (>36.2ng/L)** | **p-value** |
| --- | --- | --- | --- | --- |
|  |  |  |  |  |
| Age (years) | 65.6 (12) | 70.4 (11) | 72.4 (10.7) | <0.001 |
| Male sex | 262 (40.4%) | 375 (57.9%) | 500 (77.0%) | <0.001 |
| Race |  |  |  | <0.001 |
| -White | 375 (57.8%) | 435 (67.1%) | 498 (76.7%) |  |
| -Black | 65 (10.0%) | 56 (8.6%) | 48 (7.4%) |  |
| -Other | 209 (32.2%) | 157 (24.2%) | 103 (15.9%) |  |
| BMI (kg/m^2^) | 26.7 (23.4-30.5) | 25.9 (23.1-30.4) | 25.8 (23.2-29.2) | 0.04 |
| Smoking |  |  |  | <0.001 |
| -Current | 27 (4.2%) | 38 (5.9%) | 24 (3.7%) |  |
| -Former | 160 (24.7%) | 239 (36.9%) | 313 (48.3%) |  |
| -Never | 462 (71.2%) | 370 (57.2%) | 311 (48.0%) |  |
| Systolic BP (mmHg) | 121.5 (15.6) | 119.0 (18.3) | 118.2 (19.0) | <0.001 |
| Diastolic BP (mmHg) | 71.8 (10.2) | 68.7 (10.7) | 67.4 (11.0) | <0.001 |
| Diabetes | 226 (34.8%) | 312 (48.1%) | 344 (53.0%) | <0.001 |
| Previous stroke | 29 (4.5%) | 56 (8.6%) | 66 (10.2%) | <0.001 |
| COPD | 79 (12.2%) | 105 (16.2%) | 128 (19.7%) | 0.001 |
| Atrial fibrillation | 123 (19.0%) | 220 (34.0%) | 282 (43.5%) | <0.001 |
| NYHA class |  |  |  | <0.001 |
| -II | 266 (41.0%) | 229 (35.3%) | 184 (28.4%) |  |
| -III/IV | 383 (59.0%) | 419 (64.7%) | 465 (71.6%) |  |
| LVEF % | 31.3 (6.2) | 29.8 (7.2) | 29.6 (7.1) | <0.001 |
| Ischemic etiology | 436 (67.2%) | 485 (74.8%) | 508 (78.3%) | <0.001 |
| HF duration (years) | 3.1 (1.1-6.8) | 4.1 (1.3-7.8) | 3.8 (1.4-8.2) | 0.002 |
| Heart rate (bpm) | 72.9 (11.1) | 71.4 (11.4) | 71.8 (11.1) | 0.024 |
| Beta-blocker use | 551 (84.9%) | 551 (85.0%) | 544 (83.8%) | 0.8 |
| ACE/ARB use | 608 (93.7%) | 579 (89.4%) | 551 (84.9%) | <0.001 |
| Creatinine (mg/dl) | 1.1 (0.9-1.3) | 1.4 (1.1-1.8) | 1.8 (1.4-2.2) | <0.001 |

Values are mean (SD), median (IQR), or percentage. BMI, body mass index; BP, blood pressure; COPD, chronic obstructive pulmonary disease; LVEF, left ventricular ejection fraction.

**Table S3 Baseline characteristics of RED-HF participants with complete biomarker data by thirds of MR-proADM**

| **Characteristic** | **T1 (<0.73nmol/L)** | **T2 (0.73-1.14nmol/L)** | **T3 (>1.14nmol/L)** | **p-value** |
| --- | --- | --- | --- | --- |
|  |  |  |  |  |
| Age (years) | 65.8 (12.2) | 70.3 (11) | 73 (10) | <0.001 |
| Male sex | 326 (51.5%) | 375 (59.5%) | 403 (65.5%) | <0.001 |
| Race |  |  |  | <0.001 |
| -White | 334 (52.8%) | 449 (71.3%) | 507 (82.4%) |  |
| -Black | 88 (13.9%) | 48 (7.6%) | 30 (4.9%) |  |
| -Other | 211 (33.3%) | 133 (21.1%) | 78 (12.7%) |  |
| BMI (kg/m^2^) | 25.6 (22.6-29.3) | 26.1 (23.1-30.0) | 26.6 (24.0-30.8) | <0.001 |
| Smoking |  |  |  | <0.001 |
| -Current | 29 (4.6%) | 35 (5.6%) | 24 (3.9%) |  |
| -Former | 177 (28.0%) | 237 (37.6%) | 282 (45.9%) |  |
| -Never | 426 (67.4%) | 358 (56.8%) | 308 (50.2%) |  |
| Systolic BP (mmHg) | 121.3 (16.9) | 119.9 (17.5) | 117.4 (18.8) | <0.001 |
| Diastolic BP (mmHg) | 71.6 (10.3) | 68.6 (11.0) | 67.0 (10.6) | <0.001 |
| Diabetes | 247 (39.0%) | 295 (46.8%) | 294 (47.8%) | 0.003 |
| Previous stroke | 35 (5.5%) | 53 (8.4%) | 59 (9.6%) | 0.022 |
| COPD | 71 (11.2%) | 116 (18.4%) | 116 (18.9%) | <0.001 |
| Atrial fibrillation | 112 (17.7%) | 210 (33.3%) | 289 (47.0%) | <0.001 |
| NYHA class |  |  |  | <0.001 |
| -II | 266 (42.0%) | 232 (36.8%) | 157 (25.5%) |  |
| -III/IV | 367 (58.0%) | 398 (63.2%) | 458 (74.5%) |  |
| LVEF % | 30.3 (6.8) | 30.4 (6.8) | 30.0 (7.0) | 0.671 |
| Ischemic etiology | 416 (65.7%) | 474 (75.2%) | 489 (79.5%) | <0.001 |
| HF duration (years) | 2.7 (0.9-6.6) | 4.0 (1.5-8.2) | 4.4 (1.8-8.4) | <0.001 |
| Heart rate (bpm) | 72.6 (10.9) | 71.6 (11.2) | 71.3 (11.6) | 0.055 |
| Beta-blocker use | 527 (83.3%) | 549 (87.1%) | 513 (83.4%) | 0.097 |
| ACE/ARB use | 592 (93.5%) | 583 (92.5%) | 505 (82.1%) | <0.001 |
| Creatinine (mg/dl) | 1.0 (0.9-1.3) | 1.4 (1.1-1.7) | 1.8 (1.4-2.2) | <0.001 |

Values are mean (SD), median (IQR), or percentage. BMI, body mass index; BP, blood pressure; COPD, chronic obstructive pulmonary disease; LVEF, left ventricular ejection fraction.

**Table S4** **Baseline characteristics of RED-HF participants with complete biomarker data by thirds of copeptin**

| **Characteristic** | **T1 (<8.66pmol/L)** | **T2 (8.66-22.9pmol/L)** | **T3 (>22.99pmol/L)** | **p-value** |
| --- | --- | --- | --- | --- |
|  |  |  |  |  |
| Age (years) | 68.3 (11.7) | 68.7 (12) | 72 (10.4) | <0.001 |
| Male sex | 282 (45.1%) | 370 (59.3%) | 449 (72.0%) | <0.001 |
| Race |  |  |  | <0.001 |
| -White | 375 (60.0%) | 433 (69.4%) | 478 (76.6%) |  |
| -Black | 42 (6.7%) | 67 (10.7%) | 56 (9.0%) |  |
| -Other | 208 (33.3%) | 124 (19.9%) | 90 (14.4%) |  |
| BMI (kg/m^2^) | 26.1 (22.7-29.8) | 26.1 (23.4-30.7) | 26.0 (23.7-29.7) | 0.232 |
| Smoking |  |  |  | <0.001 |
| -Current | 27 (4.3%) | 37 (5.9%) | 24 (3.9%) |  |
| -Former | 168 (26.9%) | 232 (37.2%) | 292 (46.9%) |  |
| -Never | 430 (68.8%) | 355 (56.9%) | 306 (49.2%) |  |
| Systolic BP (mmHg) | 122.4 (16.7) | 118.8 (17.7) | 117.5 (18.7) | <0.001 |
| Diastolic BP (mmHg) | 71.6 (10.8) | 68.7 (10.5) | 67.0 (10.7) | <0.001 |
| Diabetes | 248 (39.7%) | 277 (44.4%) | 309 (49.5%) | 0.002 |
| Previous stroke | 40 (6.4%) | 40 (6.4%) | 67 (10.7%) | 0.005 |
| COPD | 91 (14.6%) | 92 (14.7%) | 119 (19.1%) | 0.049 |
| Atrial fibrillation | 141 (22.6%) | 199 (31.9%) | 269 (43.1%) | <0.001 |
| NYHA class |  |  |  | 0.007 |
| -II | 236 (37.8%) | 229 (36.7%) | 187 (30.0%) |  |
| -III/IV | 389 (62.2%) | 395 (63.3%) | 437 (70.0%) |  |
| LVEF % | 31.1 (6.5) | 29.8 (7.0) | 29.8 (7.1) | 0.002 |
| Ischemic etiology | 436 (69.8%) | 453 (72.6%) | 487 (78.0%) | 0.003 |
| HF duration (years) | 3.2 (1.1-7.6) | 3.8 (1.4-7.7) | 4.1 (1.6-7.8) | 0.015 |
| Heart rate (bpm) | 72.4 (11.2) | 71.3 (11.5) | 71.8 (11.0) | 0.191 |
| Beta-blocker use | 514 (82.2%) | 532 (85.3%) | 539 (86.4%) | 0.111 |
| ACE/ARB use | 586 (93.8%) | 557 (89.3%) | 531 (85.1%) | <0.001 |
| Creatinine (mg/dl) | 1.1 (0.9-1.3) | 1.3 (1.1-1.7) | 1.8 (1.5-2.2) | <0.001 |

Values are mean (SD), median (IQR), or percentage. BMI, body mass index; BP, blood pressure; COPD, chronic obstructive pulmonary disease; LVEF, left ventricular ejection fraction.

**Table S5 Baseline characteristics of RED-HF participants with complete biomarker data by thirds of cystatin C**

| **Characteristic** | **T1 (<1.22mg/L)** | **T2 (1.22-1.84mg/L)** | **T3 (>1.84mg/l)** | **p-value** |
| --- | --- | --- | --- | --- |
|  |  |  |  |  |
| Age (years) | 65.7 (12.4) | 70.1 (10.6) | 72.6 (10.5) | <0.001 |
| Male sex | 338 (51.5%) | 367 (57.1%) | 432 (66.8%) | <0.001 |
| Race |  |  |  | <0.001 |
| -White | 394 (60.1%) | 427 (66.4%) | 488 (75.4%) |  |
| -Black | 67 (10.2%) | 62 (9.6%) | 40 (6.2%) |  |
| -Other | 195 (29.7%) | 154 (24.0%) | 119 (18.4%) |  |
| BMI (kg/m^2^) | 26.3 (23.4-30.0) | 25.9 (23.1-30.1) | 26.1 (23.4-29.8) | 0.808 |
| Smoking |  |  |  | <0.001 |
| -Current | 31 (4.7%) | 32 (5.0%) | 26 (4.0%) |  |
| -Former | 169 (25.8%) | 242 (37.6%) | 301 (46.7%) |  |
| -Never | 456 (69.5%) | 369 (57.4%) | 318 (49.3%) |  |
| Systolic BP (mmHg) | 121.0 (16.3) | 119.6 (18.1) | 118.0 (18.7) | 0.004 |
| Diastolic BP (mmHg) | 71.8 (10.3) | 69.1 (10.7) | 67.0 (10.9) | <0.001 |
| Diabetes | 255 (38.9%) | 298 (46.3%) | 329 (50.9%) | <0.001 |
| Previous stroke | 33 (5.0%) | 51 (7.9%) | 67 (10.4%) | 0.002 |
| COPD | 81 (12.3%) | 116 (18.0%) | 115 (17.8%) | 0.007 |
| Atrial fibrillation | 139 (21.2%) | 222 (34.5%) | 264 (40.8%) | <0.001 |
| NYHA class |  |  |  | 0.001 |
| -II | 256 (39.0%) | 231 (35.9%) | 191 (29.5%) |  |
| -III/IV | 400 (61.0%) | 412 (64.1%) | 456 (70.5%) |  |
| LVEF % | 30.6 (6.5) | 30.1 (7.1) | 30.0 (7.1) | 0.469 |
| Ischemic etiology | 443 (67.5%) | 473 (73.6%) | 512 (79.1%) | <0.001 |
| HF duration (years) | 3.2 (1.1-6.9) | 3.4 (1.2-7.8) | 4.2 (1.6-8.2) | <0.001 |
| Heart rate (bpm) | 72.8 (10.6) | 72.3 (11.5) | 71.0 (11.3) | 0.006 |
| Beta-blocker use | 541 (82.5%) | 545 (84.8%) | 559 (86.4%) | 0.143 |
| ACE/ARB use | 611 (93.1%) | 591 (91.9%) | 536 (82.8%) | <0.001 |
| Creatinine (mg/dl) | 1.0 (0.9-1.3) | 1.4 (1.1-1.7) | 1.8 (1.5-2.2) | <0.001 |

Values are mean (SD), median (IQR), or percentage. BMI, body mass index; BP, blood pressure; COPD, chronic obstructive pulmonary disease; LVEF, left ventricular ejection fraction.

**Table S6** **Baseline characteristics of RED-HF participants with complete biomarker data by thirds of hsCRP**

| **Characteristic** | **T1 (<1.40mg/L)** | **T2 (1.40-4.94mg/L)** | **T3 (>4.94mg/l)** | **p-value** |
| --- | --- | --- | --- | --- |
|  |  |  |  |  |
| Age (years) | 71 (62-78) | 71 (62-78) | 71 (63-78) | 0.65 |
| Male sex | 373 (57.5%) | 348 (53.7%) | 416 (64.1%) | <0.001 |
| Race |  |  |  | <0.001 |
| -White | 408 (62.2%) | 439 (67.7%) | 462 (71.2%) |  |
| -Black | 41 (6.3%) | 65 (10.0%) | 63 (9.7%) |  |
| -Other | 200 (30.8%) | 144 (22.2%) | 124 (19.1%) |  |
| BMI (kg/m^2^) | 24.9 (22.3-27.9) | 27.0 (24.1-30.9) | 26.5 (23.7-31.2) | <0.001 |
| Smoking |  |  |  | 0.001 |
| -Current | 31 (4.8%) | 23 (3.5%) | 35 (5.4%) |  |
| -Former | 208 (32.1%) | 232 (35.8%) | 272 (42.0%) |  |
| -Never | 409 (63.1%) | 393 (60.6%) | 341 (52.6%) |  |
| Systolic BP (mmHg) | 119.9 (17.2) | 120.1 (18.1) | 118.7 (18.0) | 0.25 |
| Diastolic BP (mmHg) | 69.6 (10.8) | 69.8 (11.1) | 68.5 (10.5) | 0.035 |
| Diabetes | 283 (43.6%) | 283 (43.7%) | 316 (48.7%) | 0.11 |
| Previous stroke | 43 (6.6%) | 51 (7.9%) | 57 (8.8%) | 0.35 |
| COPD | 78 (12.0%) | 93 (14.4%) | 141 (21.7%) | <0.001 |
| Atrial fibrillation | 171 (26.3%) | 201 (31.0%) | 253 (39.0%) | <0.001 |
| NYHA class |  |  |  | <0.001 |
| -II | 255 (39.3%) | 239 (36.9%) | 184 (28.4%) |  |
| -III/IV | 394 (60.7%) | 409 (63.1%) | 465 (71.6%) |  |
| LVEF % | 30.4 (6.7) | 30.3 (6.9) | 29.9 (7.0) | 0.4 |
| Ischemic etiology | 486 (74.9%) | 468 (72.2%) | 474 (73.0%) | 0.54 |
| HF duration (years) | 3.3 (1.2-7.2) | 3.8 (1.3-7.7) | 3.6 (1.4-7.9 | 0.34 |
| Heart rate (bpm) | 71.5 (11.2) | 71.4 (10.7) | 73.2 (11.6) | 0.024 |
| Beta-blocker use | 547 (84.3%) | 561 (86.6%) | 537 (82.7%) | 0.16 |
| ACE/ARB use | 605 (93.2%) | 580 (89.5%) | 553 (85.2%) | <0.001 |
| Creatinine (mg/dl) | 1.3 (1.0-1.7) | 1.4 (1.0-1.8) | 1.5 (1.2-1.9) | <0.001 |

Values are mean (SD), median (IQR), or percentage. BMI, body mass index; BP, blood pressure; COPD, chronic obstructive pulmonary disease; LVEF, left ventricular ejection fraction.

**Table S7** **Spearman correlation (*r*) of continuous variables with each other at baseline**

|  | NT-proBNP | Troponin T | MR-proADM | Copeptin | Cystatin C | CRP |
| --- | --- | --- | --- | --- | --- | --- |
| Age  (years) | 0.188 (p<0.001) | 0.239  (p<0.001) | 0.256  (p<0.001) | 0.130  (p<0.001) | 0.241  (p<0.001) | 0.008  (p=0.71) |
| BMI  (kg/m^2^) | -0.203  (p<0.001) | -0.057  (p<0.001) | 0.109  (p<0.001) | 0.021  (p=0.37) | -0.002  (p=0.92) | 0.158  (p<0.001) |
| Systolic BP  (mmHg) | -0.154  (p<0.001) | -0.083  (p<0.001) | -0.096  (p<0.001) | -0.120  (p<0.001) | -0.076  (p=0.001) | -0.030  (p=0.19) |
| Diastolic BP  (mmHg) | -0.175  (p<0.001) | -0.173  (p<0.001) | -0.183  (p<0.001) | -0.174  (p<0.001) | -0.188  (p<0.001) | -0.047  (p=0.037) |
| LVEF  (%) | -0.203  (p<0.001) | -0.088  (p<0.001) | -0.013  (p=0.56) | -0.069  (p=0.003) | -0.027  (p=0.23) | -0.025  (p=0.28) |
| HF duration  (years) | 0.041  (p=0.071) | 0.068  (p=0.003) | 0.135  (p<0.001) | 0.066  (p=0.004) | 0.088  (p<0.001) | 0.033  (p=0.15) |
| Heart rate  (bpm) | -0.013  (p=0.58) | -0.042  (p=0.064) | -0.055  (p=0.017) | -0.014  (p=0.54) | -0.072  (p=0.001) | 0.043  (p=0.055) |
| Creatinine  (mg/L) | 0.366  (p<0.001) | 0.515  (p<0.001) | 0.557  (p<0.001) | 0.520  (p<0.001) | 0.597  (p<0.001) | 0.113  (p<0.001) |
| Haemoglobin  (g/dL) | -0.140  (p<0.001) | -0.108  (p<0.001) | -0.147  ( p<0.001) | -0.086  ( p<0.001) | -0.114  ( p<0.001) | -0.047  (p=0.036) |
| NT-proBNP  (ng/L) |  | 0.535  (p<0.001) | 0.460  (p<0.001) | 0.360  (p<0.001) | 0.414  (p<0.001) | 0.171  (p<0.001) |
| Troponin T  (ng/L) |  |  | 0.509  (p<0.001) | 0.449  (p<0.001) | 0.5 00  (p<0.001) | 0.178  (p<0.001) |
| MR-proADM  (nmol/L) |  |  |  | 0.497  (p<0.001) | 0.577  (p<0.001) | 0.240  (p<0.001) |
| Copeptin  (pmol/L) |  |  |  |  | 0.437  (p<0.001) | 0.146  (p<0.001) |
| Cystatin C  (mg/L) |  |  |  |  |  | 0.225  (p<0.001) |

BP, blood pressure; LVEF, left ventricular ejection fraction; NT-proBNP, N-terminal pro-B-type natriuretic peptide; MR-proADM, mid-regional pro-adrenomedullin; CRP, C-reactive protein.

**Table S8 Baseline characteristics of RED-HF participants with complete biomarker data by whether or not all-cause mortality occurred during follow-up**

| Characteristic | Deceased (any cause)  (n=932) | Not deceased (any cause)  (n=1346) |  |
| --- | --- | --- | --- |
| Age (years) | 72.4 (10.8) | 68 (11.5) | <0.001 |
| Male sex | 620 (66.5%) | 714 (53.0%) | <0.001 |
| Race  -White  -Black  -Other | 718 (77.0%)  65 (7.0%)  149 (16.0%) | 831 (61.7%)  378 (10.2%)  137 (28.1%) | <0.001 |
| BMI (kg/m^2^) | 25.6 (22.9-29.2) | 26.7 (23.7-30.4) | <0.001 |
| Smoking  -Current  -Former  -Never | 44 (4.7%)  405 (43.6%)  480 (51.7%) | 53 (3.9%)  442 (32.8%)  851 (63.2%) | <0.001 |
| Systolic BP (mmHg) | 117.6 (18.3) | 121.7 (17.5) | <0.001 |
| Diastolic BP (mmHg) | 67.1 (10.8) | 71.0 (10.6) | <0.001 |
| Diabetes | 427 (45.8%) | 628 (46.7%) | 0.692 |
| Previous stroke | 87 (9.3%) | 92 (6.8%) | 0.029 |
| COPD | 194 (20.8%) | 173 (12.9%) | <0.001 |
| Atrial fibrillation/flutter | 409 (43.9%) | 138 (25.1%) | <0.001 |
| NYHA class  -II  -III/IV | 243 (26.1%)  689 (73.9%) | 548 (40.7%)  798 (59.3%) | <0.001 |
| LVEF % | 29.5 (7.0) | 30.8 (6.7) | <0.001 |
| Ischemic etiology | 722 (77.5%) | 939 (69.8%) | <0.001 |
| HF duration (years) | 4.4 (1.7-8.3) | 3.1 (1.1-7.2) | <0.001 |
| Heart rate (bpm) | 72.0 (11.6) | 71.9 (10.8) | 0.866 |
| Beta-blocker use | 761 (81.7%) | 1176 (87.4%) | <0.001 |
| ACE-I or ARB use | 813 (87.2%) | 1212 (90.0%) | 0.036 |
| Creatinine (mg/dl) | 1.5 (1.2-2.0) | 1.3 (1.0-1.7) | <0.001 |
| NT-proBNP (ng/L) | 3123 (1430-6668) | 1130 (356-2754) | <0.001 |
| Troponin-T (ng/L) | 36.6 (22.5-54.1) | 19.7 (11.8-32.9) | <0.001 |
| MR-proADM (nmol/L) | 1.1 (0.8-1.5) | 0.8 (0.6-1.1) | <0.001 |
| Copeptin (pmol/L) | 20.7 (9.3-37.5) | 11.3 (5.5-22.8) | <0.001 |
| Cystatin C (mg/L) | 1.9 (0.7) | 1.4 (0.6) | <0.001 |
| CRP (mg/L) | 3.4 (1.3-8.3) | 2.2 (0.9-5.7) | <0.001 |

Values are mean (SD), median (IQR) or percentage. BMI, body mass index; BP, blood pressure; COPD, chronic obstructive pulmonary disease; LVEF, left ventricular ejection fraction; NT-proBNP, N-terminal pro-B-type natriuretic peptide; MR-proADM, mid-regional pro-adrenomedullin; CRP, C-reactive protein.
